# Closed and open state dependent block of potassium channels cause opposing effects on excitability – a computational approach

**DOI:** 10.1038/s41598-019-44564-x

**Published:** 2019-06-03

**Authors:** Richard Ågren, Johanna Nilsson, Peter Århem

**Affiliations:** 1 Department of Clinical Neuroscience, Karolinska Institutet, 171 76 Stockholm, Sweden; 2 Department of Medical Sciences, University of Örebro, 701 82 Örebro, Sweden; 3 Department of Neuroscience, Karolinska Institutet, 171 77 Stockholm, Sweden

**Keywords:** Biophysical methods, Ion channels in the nervous system

## Abstract

Block of voltage-gated potassium (Kv) channels has been demonstrated to affect neuronal activity described as increasing excitability. The effect has been associated with a closed-state dependent block. However, the block of Kv channels in e.g. local anesthetic and antiarrhythmics, is open state-dependent. Since the reduced excitability in this case mainly is due to sodium channel block, the role of the Kv channel block is concealed. The present investigation aims to analyse the specific role of state-dependent Kv channel block for excitability. Using a computational approach, with introduced blocked states in the Kv channel of the Frankenhaeuser-Huxley axon membrane model, we calculated the effects on threshold, firing and presynaptic Ca influx. The Ca influx was obtained from an N-type Cav channel model linked to the Frankenhaeuser-Huxley membrane. The results suggested that a selective block of open Kv channels decreased the rate of repetitive firing and the consequent Ca influx, thus challenging the traditional view. In contrast, presence of a closed-state block, increased the firing rate and the Ca influx. These findings propose that Kv channel block may either increase or decrease cellular excitability, thus highlighting the importance of further investigating the role of state-specific blocking mechanisms.

## Introduction

Voltage-gated ion channels are the main targets for a plethora of highly versatile and clinically important drugs (e.g. local anesthetics, antiarrhythmics and antiepileptics) and for an even higher number of natural toxins (e.g. tetrodotoxin, saxitoxin, batrachotoxins, dendrotoxins and charybdotoxins). These drugs and toxins often target voltage gated potassium (Kv) channels and have been proposed to exert their action by modulating nerve or muscle membrane excitability^1^. The concept of excitability may refer to different aspects in different settings. A lowered stimulation threshold, or an increased firing frequency, or a prolonged action potential waveform at the presynaptic membrane, or an increased transmitter release at the downstream synapse are often used as markers of increased neural excitability. However, some of these markers may be expected to counteract each other with respect to excitability. Previously, the effect of specific Kv channel active drugs have been demonstrated to depend on the mechanism of action^2^, with different state-dependent blocking mechanisms decreasing or increasing firing frequencies.

In the present investigation, using a computational approach, the aim is to extend previous findings and to clarify how different mechanisms of Kv channel block influence excitability in terms of effect on stimulation threshold, on action potential waveform and on firing frequency in a neuronal model. Furthermore, the resulting effects on downstream synapses are simulated by integrating the induced currents through N-type voltage-gated calcium (Cav) channels. With channel block we here refer to a mechanism equivalent to occluding the channel pore^3^. Considering the molecular structure of known Kv channels^4^, occluding the channel from the external side implies that the channel is blocked in resting closed state, and occluding the channel from the internal side means that the channel is blocked in open state. In addition to the numerical calculations of the different excitability measures we also tried to explain the results in terms of modified current-voltage relations.

There is strong experimental evidence supporting that Kv channel specific blockers increase spontaneous activity, thereby inducing increased neuronal excitability, e.g. the small-molecule Kv channel blockers 4-aminopyridine (4-AP) and tetraethylammonium (TEA) salts have been shown to be epileptogenic in mammalian nervous systems^5–8^. Similarly, several Kv channel specific peptide and protein toxins from so evolutionary diversified organisms as snakes, scorpions, sea anemones, and cone snails have been shown to be epileptogenic^9,10^. Many of these cases have been shown to concern a resting closed state binding mechanism. Thus, 4-AP has been suggested to interact with Kv1 channels by first binding in an open state and then inducing a bound closed state^11^. This would render functional effects corresponding to a closed state dependent block. An alternative model suggests 4-AP binding in both closed and open state^12^, also rendering the resulting block functionally closed-state dependent^11,12^. TEA salts have both an external state independent binding site and an internal open state dependent site in myelinated axons, suggesting binding in resting state^13^. Dendrotoxins block Kv1 channels in a manner suggesting binding in resting state^14^.

We have in previous investigations suggested that blocking Kv channels in open state may modulate the action potential train and reduce the firing frequency^2^. Assuming an open-state binding mechanism in these cases could theoretically allow for a synergism between the local anesthetic action on voltage-gated sodium (Nav) and Kv channels. For local anesthetics this assumption of open-state Kv channel blocking mechanisms has experimental support^15,16^.

To investigate the influence of different Kv channel blocking mechanisms on the excitability, we attempted a computational approach simulating the well-established membrane model of Frankenhaeuser and Huxley^17^. This model is an offspring of the traditional Hodgkin-Huxley model, describing the features of a myelinated axon, using the Goldman-Hodgkin-Katz permeability concept^18^. Through introduction of closed- and open-state blocked Kv1 channel states into the Frankenhaeuser-Huxley model and linking the model to a Cav channel^19^, the implications of differential state-dependent Kv channel effects on the neuronal firing pattern and presynaptic Ca-influx was simulated, allowing for approximation of the cumulative consequence of the firing pattern on the cellular excitability.

## Results

### Modulation of firing pattern by state-dependent Kv channel block

**Figure 1:**
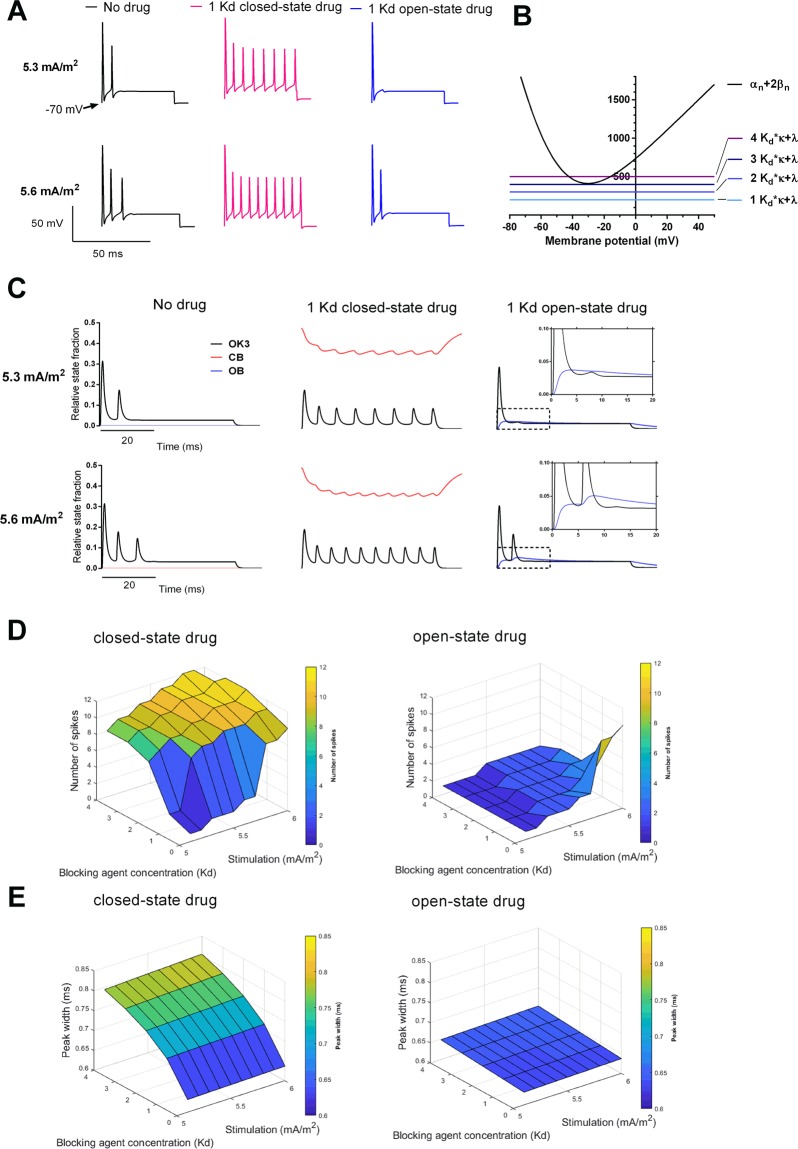
Implications of differential state-dependent Kv channel block on the firing pattern. (**A**) Spiking patterns at 5.3 and 5.6 mA/m^2^ assuming no drug or 200 μM (corresponding to 1 K_d_ concentration) on a closed state binding model (red) and an open state binding model (blue). (**B**) Potassium channel closing (α_n_+2β_n_) and blocking rates (L_o_ · κ + λ) for 1–4 K_d_ equivalents as functions of membrane potential. (**C**) Potassium channel state fractions OK3 (black), CB (red) and OB (blue) at 5.3 and 5.6 mA/m^2^ assuming no drug or 200 μM (corresponding to a concentration of 1 K_d_ equivalent) on a closed state binding model and an open state binding model. Insets for the open state binding model demonstrate the accumulation of OB over time. (**D**) Number of action potentials (above −10 mV) during 60 ms at 5.1–6.0 mA/m^2^ stimulation for 0–800 μM (corresponding to 0–4 K_d_ equivalents) on a closed state binding model and an open state binding model. (**E**) Peak width of first action potential at −10 mV at 5.1–6.0 mA/m^2^ stimulation for 0–800 μM (corresponding to 0–4 K_d_ equivalents) on a closed state binding model and an open state binding model.

The Frankenhaeuser-Huxley model responds to supra-threshold stimulation by repetitive high-frequency firing and is classified as representing Class 2 excitability in Hodgkin’s nomenclature^20^ or Type 2 excitability in the nomenclature of Robinson *et al*.^21^. In Fig. 1A, the firing pattern of this model at near threshold stimulation values (5.3 and 5.6 mA/m^2^) is compared to that of the adapted model, incorporating binding to the closed or open states of Kv channels in the presence of 200 µM blocking agent. Figure 1A shows the effect on the spiking of the adapted model assuming exclusively closed state binding at a K_d_ value of 200 µM and of the adapted model assuming exclusively open state binding at a K_d_ value of 200 µM compared to the control case. The control case induced a near-threshold spiking behavior, characteristic for Class 2 dynamics (close to an Andronov-Hopf bifurcation), comprising a few action potentials. Closed state binding induced sustained spiking, while open state binding eliminated the last action potentials. Stability of the model was assessed by analyzing the resting membrane potential after the action potential train for all combinations of closed- and open-state blocking drugs between 5.1–6.0 mA/m^2^. In all cases, the resting membrane potential returned to −70 mV when stimulation was abolished.

For open channel (use-dependent) block, the rate of channel closing (i.e. α + 2β) vs. rate of transition between open and blocked states (i.e. L_o_ × κ + λ, where L_o_ is the drug concentration) critically influences the channel behavior. In Fig. 1B the rates are plotted as functions of voltage at different concentrations. The data shows that at a drug concentration of 1 to 3 K_d_ equivalents, the open channel state (OK3) predominantly transitions into the closed channel states (CK1 and CK2) when the membrane is repolarizing. At drug concentrations above 3 K_d_ equivalents, the rate of the transitions between open and blocked states is greater than the rate of closing the channel (L_o_ × κ + λ > α_n_ + 2β_n_) between −44 to −13 mV, suggesting increased transitions from the open state (OK3) into the open-blocked state (OB) rather than to the closed channel states (CK1 and CK2). This would allow for the channel displaying burst behavior with extended time spent in the closed state.

Further information on the mechanisms of the differential effect of blocking the Kv channel in open and closed state can be obtained from the distribution of channels in open (OK3), closed-blocked (CB) and open-blocked (OB) states at various stimulating currents and drug concentrations. In Fig. 1C, the distributions at 5.3 and 5.6 mA/m^2^ stimulation and a concentration of 1 K_d_ equivalent are shown. The triggering of action potentials depends critically on the opposing forces of the K current and the Nav channel inactivation process.

For the closed-state block case the fraction of channels in open state (OK3) is markedly reduced compared to the no drug situation. The opposing effect of an increased Nav channel inactivation (not shown) was not sufficiently strong to overcome the effect of the K current, thus rendering a lowered impulse generating threshold, which induces spiking in accordance with the results in Fig. 1A.

For the open-state block case, on the other hand, the decreased K current was not sufficient to overcome the enhanced Nav channel inactivation, leading to an increased triggering threshold and a block of the impulse generation in accordance with the results in Fig. 1A. Parallel with these processes was an accumulation of channels in open-blocked state (OB), followed by a slow decrease over time.

This differential response to the two cases of state-specific binding, measured as number of action potentials per stimulation step duration (50 ms), was consistent over a range of stimulation levels for different concentrations (Fig. 1D). Whereas increased closed state Kv channel block increased the number of action potentials for all stimulations, a decrease was noticed for the open state Kv channel block. The induced prolongation of the action potential was present over a range of stimulation levels for both the closed state binding and the open state binding cases (Fig. 1E). The absolute increase in peak width for the closed state Kv channel block was pronounced (25% at 800 µM as compared to no drug) as compared to the open state Kv channel block (2.5% at 800 µM as compared to no drug).

### Modulation of presynaptic Ca influx by state-dependent Kv channel block

**Figure 2:**
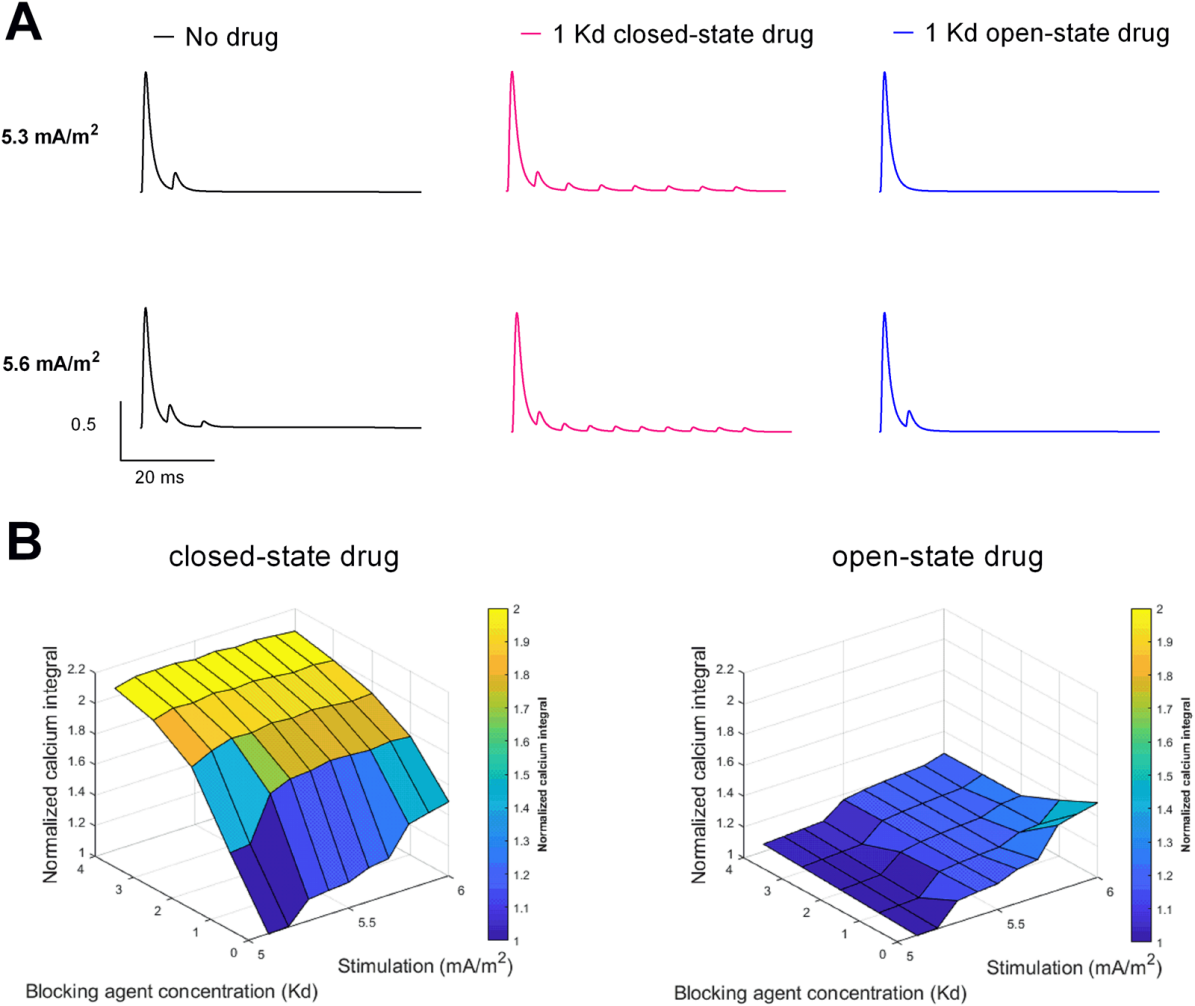
Normalized Ca influx for closed state and open state binding of Kv blocking drug. (**A**) Normalized Ca currents at 5.3 and 5.6 mA/m^2^ assuming no drug (black) or 200 μM (corresponding to 1 K_d_ concentration) on a closed-state binding model (red) and an openstate binding model (blue). (**B**) Normalized Ca integral at 5.1–6.0 mA/m^2^ stimulation for 0–800 μM (corresponding to 0–4 K_d_ equivalents; K_d_ = 200 μM) drug concentration for the closed state and open state binding cases.

The implications of the firing pattern modulations on the excitability on downstream synaptic activity were analysed by modelling the Ca influx through a hypothetical synaptic N-type Cav channel connected to Frankenhaeuser-Huxley membrane model. Using the previous stimulation and drug protocol, the repetitive spiking for both 5.3 and 5.6 mA/m^2^ was evident for the closed state binding case, whereas the reduced number of spikes was evident for the open state binding case (Fig. 2A). The Ca influx was normalized over time to provide an integral over a range of stimulation levels for different concentrations of the blocking drug (Fig. 2B). An increase in Ca influx was noticed for all concentrations and all stimulation levels analysed for the closed state binding case, whereas for the open-state binding case a reduced Ca influx was noticed for corresponding concentrations and stimulation levels.

### Analysis of excitability limit following state-dependent Kv channel block

**Figure 3:**
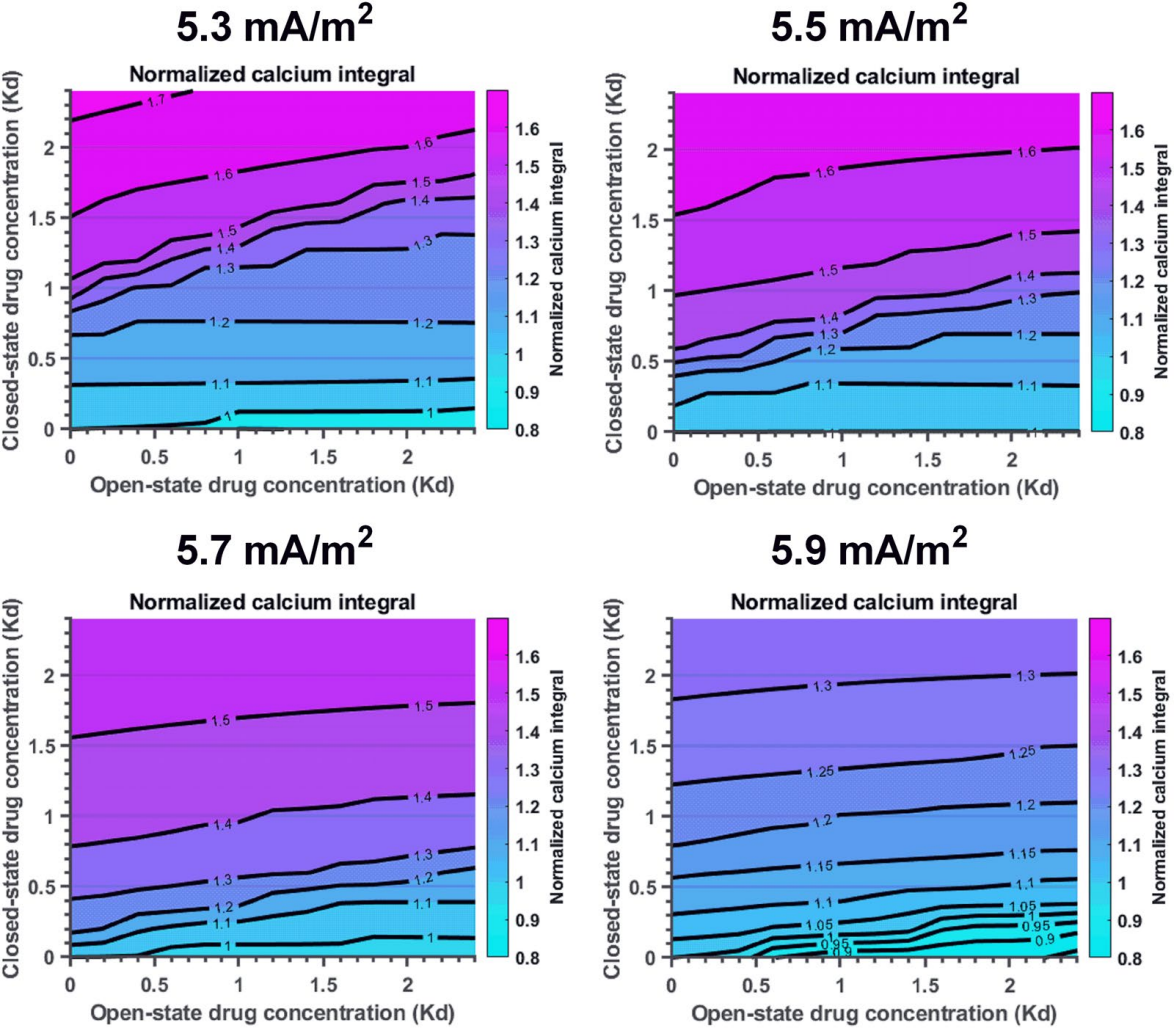
Normalized calcium integrals for different stimulations depending on the affinity of the resting and the open state for a Kv specific drug. Stimulations from 5.3 to 5.9 mA/m^2^. Drug concentrations from 0–480 μM (corresponding to 0–2.4 K_d_). The dominant effect is that of an increased normalized calcium integral (>1).

The question how the relation between open and closed state affinity for a Kv channel blocking drug affects the influence of the drug on excitability was analysed by simulating different degrees of closed state and open state binding simultaneously. Figure 3 shows results from computations at stimulations between 5.3 and 5.9 mA/m^2^. Stability of the model was assessed by analyzing the resting membrane potential after the action potential train for all combinations of closed- and open-state blocking drugs at 5.3–5.9 mA/m^2^. In all cases, the resting membrane potential returned to −70 mV. Large areas of the contour plots depicted an increased Ca integral (>1), while areas depicting an integral <1 were smaller and only noticed at 5.3, 5.7 and 5.9 mA/m^2^ stimulation. Nevertheless, the present findings suggest that open-state Kv channel binding could reduce synaptic Ca influx and postsynaptic excitability. The border between areas depicting an increased and a decreased Ca integral was consistently requiring a ratio between the K_d_ for the closed state binding and the K_d_ for the open state binding of around 6–12.

## Discussion

The background to the present study was the widely held belief that pharmacological blocking or genetic elimination of Kv channels in neurons generally induce an increased excitability^5–7^. This would leave us with a paradoxical situation with regards to local anesthetics that block both Nav and Kv channels, with the action on Kv channels opposing the inhibiting effect of the Nav channel block. We therefore found it of interest to analyse how the state dependency of the binding affected the effect on the excitability.

The effects of Kv channels in which the pore was occluded in the resting or closed channel state were investigated using the experimentally based computational membrane model of Frankenhaeuser and Huxley, extended by the blocked resting and open states, and linked to the Cav channel model of Patil *et al*.^19^. The results confirmed that, and detailed how, the hypothetical drug binding to closed Kv channels increased the excitability, measured as a decreased threshold for the stimulation current, an increased firing frequency and an increased Ca inflow at the downstream presynaptic membrane. In contrast, hypothetical drug binding solely to open Kv channels decreased the excitability measures, caused an increased current threshold, a decreased firing frequency and a decreased Ca inflow. Furthermore, assuming binding to both resting and open channels, it was demonstrated that the binding affinity in open state had to be approximately 6–12-fold more potent as compared to that in the resting state in order to cause a reduced excitability.

Information on the mechanisms behind the effects on the excitability measures of the state-specific Kv channel block were obtained from the analysis of the distribution of open, closed-blocked and open-blocked Kv channel states during the action potential train, shown in Fig. 1C. The cause of the differential effects of closed and open state binding was found to be the critical relation between of the opposing actions of the K current and the Nav channel inactivation.

For the closed channel block case, the maximum number of channels in conducting state (OK3) was markedly reduced compared to the no drug case along with a reduction of the number of channels in closed-blocked state (CB) during the stimulation. An increased fraction of channels in closed-state block shifts the balance between channels in closed state (CK2) and closed-blocked state (CB) towards the latter, further decreasing the fraction of channels in conducting state (OK3). The increase of the Nav channel inactivation was not sufficient to counteract the reduction of the K current, resulting in repetitive spiking.

For the open channel block case, in contrast, the reduction of the K current was not sufficient to counteract the increased Nav channel inactivation, resulting in a block of impulse generation^22^. An accumulation of channels in the open-blocked state (OB) followed by a gradual reduction suggested a foot-in-the-door mechanism.

Antiarrhythmic drugs of class III inhibit outward potassium currents, increasing the refractory period of cardiomyocytes and suppressing atrial arrhythmias^23^. However, based on the prevalent side effect of the ventricular tachyarrhythmia torsades de pointes^24^, the interest for subtype- and state-specific potassium channel blocking agents has increased. Computational investigations suggested that targeting open- and inactivated-states of the cardiac Kv1.5 channels to be beneficial for antiarrhythmic drug action as compared to the closed Kv1.5 channel state^25,26^. In previous investigations of cardiac Kv4.2/4.3 channels, closed- and, more so, open-state channel block prolonged the action potential durations, although with varying potencies, depending on the fraction of repolarization used to assess the action potential durations^27^. Our results using the neuronal firing model suggest prolongation of the action potential peak widths following both closed- and open-state Kv channel block, with approximately a tenfold prolongation by the former. These findings highlight the discrepancies resulting from state-dependent Kv channel block possibly present in neurons.

The present simulations are based on binding constants estimated from electrophysiological studies of the local anesthetic drug bupivacaine on Kv1.1 channels^15^, which limits the direct generalizability of the findings. In addition, there are about 40 different Kv channel genes in the human nervous system^28^, and each neuron comprises several types of heteromeric Kv channels, presumably with different affinities in different states for different drugs and toxins. A similar channel diversity is noted for cardiac ion channels^29,30^, complicating accurate *in silico* modelling and discovery of channel-specific therapeutic drugs.

However, in conclusion, the present computational investigation is challenging the common notion that Kv channel blockers per se are proexcitatory. Rather, it stresses the importance of analyzing state dependency of binding when developing drugs with significant affinity to Kv channels. It also stresses the importance of analyzing the state dependency when trying to understand the mechanisms underlying the clinical symptoms of natural toxins as many of them specifically target Kv channels, and thus have been suggested to account for over 150 000 deaths annually^31^. Numerous details regarding their pharmacology remain to be revealed.

## Methods

### Nodal membrane model

The Frankenhaeuser-Huxley model is based on voltage-clamp data from sciatic nerves from the African clawed toad (*Xenopus laevis*)^17^. To model the action potential train, the time-derivate of the membrane potential V was numerically calculated

$$\frac{dV}{dt}=\frac{{I}_{stim}-{I}_{Na}({O}_{Na})-{I}_{K}({O}_{K})-{I}_{leak}(V)}{{C}_{M}}$$

where I_stim_ denotes the stimulating current, I_Na_, I_K_ and I_leak_ denote sodium, potassium and leak currents, O_Na_, O_K_ are open probabilities derived from the Markov schemes described below and C_M_ the membrane capacitance. The Goldman-Hodgkin-Katz permeability equation determines I_Na_ and I_K_

$$\begin{array}{ccc}{I}_{Na} & = & {O}_{Na}{\bar{P}}_{Na}VF\zeta \frac{{[Na]}_{o}-{[Na]}_{i}\exp (V\zeta )}{1-\exp (V\zeta )}\\ {I}_{K} & = & {O}_{K}{\bar{P}}_{K}VF\zeta \frac{{[K]}_{o}-{[K]}_{i}\exp (V\zeta )}{1-\exp (V\zeta )}\end{array}$$

where Pbar_Na_ and Pbar_K_ denote sodium and potassium channel permeability constants, R, T and F denote the gas constant, the thermodynamic temperature and the Faraday constant, respectively. *ζ* = F/RT. [Na]_o_, [Na]_i_, [K]_o_ and [K]_i_ denote external and internal concentrations of sodium and potassium ions respectively. The leak current is defined by

$${I}_{leak}={g}_{leak}(V-{E}_{leak})$$

where g_leak_ denotes the leak conductance and E_leak_ the equilibrium value for the leak current. Parameter values are listed in Table 1.

**Table 1:** Parameter values for the neuronal firing model.

Parameter	Value
T	295 K
[Na]_i_	15 M m^−3^
[Na]_o_	115 M m^−3^
[K]_i_	120 M m^−3^
[K]_o_	2.5 M m^−3^
V_leak_	−7 * 10^−2^ V
G_leak_	300 S m^−2^
C_m_	2 * 10^−2^ F m^−2^
Pbar_Na_	8 * 10^−5^ m s^−1^
Pbar_K_	1.2 * 10^−5^ m s^−1^
κ	5 * 10^5^ s^−1^ M^−1^
λ	100 s^−1^

### Nav channel model

**Figure 4:**
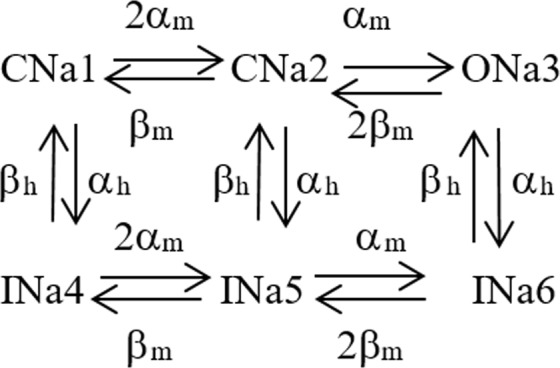
Markov model of Nav channel derived from the Frankenhaeuser-Huxley model. CNa_j_, ONa_j_ and INa_j_ represent closed, open and inactivated channel states and α_j_ and β_j_ voltage-dependent rate constants.

The Nav channel model was derived from the Frankenhaeuser-Huxley model^17^. The model can be described as a Markov model (Fig. 4).

The corresponding equation system to be solved can be expressed in matrix form as

$$\frac{{\rm{d}}({O}_{Na})}{{\rm{dt}}}=[\begin{array}{c}{C}_{Na1}\\ {C}_{Na2}\\ {O}_{Na3}\\ {I}_{Na4}\\ {I}_{Na5}\\ {I}_{Na6}\end{array}][\begin{array}{cccccc}-2{\alpha }_{m}-{\alpha }_{h} & +{\beta }_{m} & 0 & +{\beta }_{h} & 0 & 0\\ +2{\alpha }_{m} & -({\beta }_{m}+{\alpha }_{m}+{\alpha }_{h}) & +2{\beta }_{m} & 0 & +{\beta }_{h} & 0\\ 0 & +{\alpha }_{m} & -(2{\beta }_{m}+{\alpha }_{h}) & 0 & 0 & +{\beta }_{h}\\ +{\alpha }_{h} & 0 & 0 & -{\beta }_{h} & 0 & 0\\ 0 & +{\alpha }_{h} & 0 & 0 & -{\beta }_{h} & 0\\ 0 & 0 & +{\alpha }_{h} & 0 & 0 & -{\beta }_{h}\end{array}]$$

The voltage-dependent rate constants were defined by the following equations:

$$\,\begin{array}{rcl}{\alpha }_{m}(V) & = & \frac{360000(V+0.048)}{1-\exp \,[-\frac{(V+0.048)}{0.003}]}\\ {\beta }_{m}(V) & = & \frac{-400000(V+0.057)}{1-\exp \,[\frac{(V+0.057)}{0.02}]}\\ {\alpha }_{h}(V) & = & \frac{-100000(V+0.08)}{1-\exp \,[\frac{(V+0.08)}{0.006}]}\\ {\beta }_{h}(V) & = & \frac{4500}{1+\exp \,[-\frac{(V+0.025)}{0.01}]}\end{array}$$

### Kv channel block models

**Figure 5:**
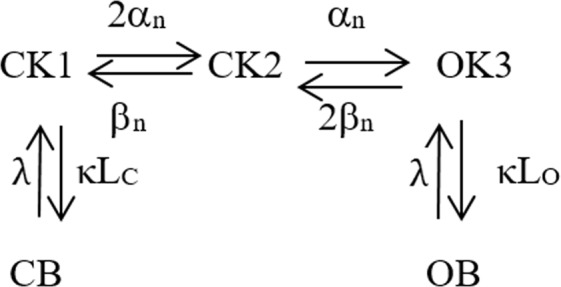
Markov model of Kv channel derived from the Frankenhaeuser-Huxley model with introduced closed and open blocked states, CB and OB. α_j_ and β_j_ denote voltage-dependent rate constants. κ and λ denote binding rate constants, L_C_ and L_O_ the concentration of the closed and open state blocking agent respectively.

For the Kv channel, closed- and open-blocked states were introduced in addition to the original Frankenhaeuser-Huxley model^17^, yielding the following Markov scheme (Fig. 5).

The corresponding equation system can be expressed in matrix form as

$${\rm{d}}({O}_{K})/{\rm{dt}}=[\begin{array}{c}{C}_{K1}\\ {C}_{K2}\\ {O}_{K3}\\ OB\\ CB\end{array}][\begin{array}{ccccc}-(2{\alpha }_{n}+{\rm{\kappa }}{L}_{C}) & +{\beta }_{n} & 0 & 0 & +{\rm{\lambda }}\\ +2{\alpha }_{n} & -({\beta }_{n}+{\alpha }_{n}) & +2{\beta }_{n} & 0 & 0\\ 0 & +{\alpha }_{n} & -(2{\beta }_{n}+{\rm{\kappa }}{L}_{O}) & +{\rm{\lambda }} & 0\\ 0 & 0 & +{\rm{\kappa }}{L}_{O} & -{\rm{\lambda }} & 0\\ +{\rm{\kappa }}{L}_{C} & 0 & 0 & 0 & -{\rm{\lambda }}\end{array}]$$

The voltage dependent rate constants were defined by the following equations:

$$\begin{array}{rcl}{\alpha }_{n}(V) & = & \frac{20000(V+0.035)}{1-\exp [-\frac{(V+0.035)}{0.01}]}\\ {\beta }_{n}(V) & = & \frac{-50000(V+0.06)}{1-\exp [\frac{(V+0.06)}{0.01}]}\end{array}$$

The Kv channel binding rates, for the CB and OB states, e.g. κ and λ, were derived from previously described simulated voltage-clamp data of local anesthetic block on Kv1.1 channels^32^, and are presented in Table 1 below. The K_d_ value (λ/κ) was 200 µM. It should be noted that only the binding rate constants for the open state case were estimated from the experimental recordings^32^, and that the corresponding rate constants for closed state binding were assumed to equal those of the open state case.

### N-type Cav channel model

**Figure 6:**
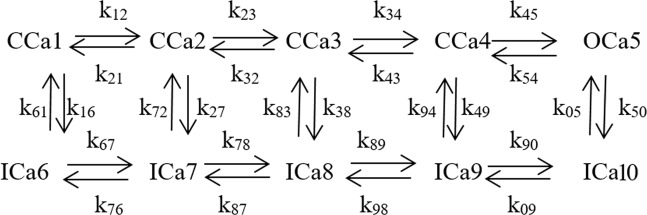
N-type Cav channel model derived from Patil *et al*. CCa_j_, OCa_j_ and ICa_j_ represent closed, open and inactivated states, k_ij_ rate constants.

To analyse the implications of the neuronal firing pattern modulation, by state-specific Kv channel blocking mechanisms, on the presynaptic Ca influx, an N-type Cav channel was linked to the Frankenhaeuser-Huxley model and simulated according to the description of the intermediate closed-state inactivating model of Patil *et al*.^19^ (Fig. 6).

The corresponding equation system can be expressed in matrix form as:1The transition between CCa4 and OCa5, and between OCa5 and ICa10, as well as the transitions between closed and inactivated states were assumed to be voltage independent, with values given in Table 2 below. The voltage dependent rate constants were given by

$$k={k}_{0}\exp [\frac{z\,\ast \,e(\delta V-{V}_{0})}{{k}_{B}\,\ast \,T}]$$

where k_o_ is constant (in units of s^−1^), z is the valence of the gating charge associated with the transition, e is an elementary charge, V is membrane voltage, δ is the fraction of gating charge that is moved to reach the transition state, V_o_ is offset voltage, and k_B_ is the Boltzmann constant, see Table 2 derived from Patil *et al*.^19^. The use of the closed-state inactivating Cav channel model of Patil *et al*.^19^ allowed for simulations of high frequency Ca influx, as compared to previous, open-state inactivating, Cav models^33^.

**Table 2:** Parameter values for the N-type Cav channel model.

Voltage-dependent rates				
	k_o_ (s^−1^)	z	δ	V_o_ (V)
k_12_	9 × 10^2^	+2	0.3	0
k_21_	9 × 10^2^	−2	0.7	−4.5 × 10^−2^
k_23_	1.5 × 10^3^	+1.5	0.95	0
k_32_	1.5 × 10^3^	−1.5	0.05	−3 × 10^−2^
k_34_	1 × 10^3^	+2.36	0.7	1 × 10^−3^
k_43_	1 × 10^3^	−2.36	0.3	−1 × 10^−3^
k_67_	6.3 × 10^5^	+2	0.3	0
k_76_	9 × 10^2^	−2	0.7	−4.5 × 10^−2^
k_78_	1.2 × 10^4^	+1.5	0.95	0
k_87_	1.5 × 10^3^	−1.5	0.05	−3 × 10^−2^
k_89_	1 × 10^3^	+2.36	0.7	1 × 10^−3^
k_98_	1 × 10^3^	−2.36	0.3	−1 × 10^−3^
**Voltage-independent rates (s** ^**−1**^ **)**				
k_16_	10^−1^			
k_61_	5.6 × 10^−1^			
k_27_	1.25 × 10^1^			
k_72_	10^0^			
k_38_	5 × 10^1^			
k_83_	5 × 10^−1^			
k_49_	3 × 10^0^			
k_94_	3 × 10^−2^			
k_50_	2 × 10^0^			
k_05_	2 × 10^−2^			
k_45_	10^3^			
k_54_	10^3^			
k_90_	10^3^			
k_09_	10^3^			

### Simulation of spiking in the nodal membrane model

Initially, the pulse current model was equilibrated at a holding potential of −70 mV, with no stimulating current for 50 ms. Production runs of 60 ms stimulation, using pulse currents between 5.1 to 6 mA/m^2^, followed by 10 ms with no stimulating current, were calculated at different concentrations of blocking agents. The integral of Ca current over 70 ms was calculated for comparison between different levels of Kv channel block. The timesteps for all calculations were 5 µs. All simulations were conducted using Matlab R2017a software. Graphical objects were created in GraphPad Prism 6.

## Data Availability

The datasets generated during and/or analysed during the current study are available from the corresponding author on reasonable request.
